# Special Issue: “Anti-inflammatory Effects of Glucagon-like Peptide-1”

**DOI:** 10.3390/ijms25041997

**Published:** 2024-02-07

**Authors:** Alessandra Puddu, Davide Maggi

**Affiliations:** Department of Internal Medicine and Medical Specialties, University of Genoa, 16132 Genoa, Italy; davide.maggi@unige.it

From the failure of gut extracts in diabetic patients’ therapy to the effective action in cardiovascular outcomes [1,2], the story of GLP-1 in type 2 diabetes is intriguing and still developing.

The sequence of glucagon-like peptide-1 (GLP-1) was described in the early 1980s with the cloning of the cDNAs and genes encoding proglucagon [3,4,5,6]. GLP-1 is a single glycosylated polypeptide of 30 amino acids derived from the post-translational processing of proglucagon by the prohormone convertase 1 (PC1/3) [7]. The biologically active forms of GLP-1 are GLP-1-(7-37) and GLP-1-(7-36)NH2, which are mainly produced by enteroendocrine L cells. Studies on the physiological role of GLP-1 in humans have shown that it is secreted after nutrient intake and stimulates insulin secretion in the presence of hyperglycemia [8]. Therefore, GLP-1 is defined as an incretin hormone.

The biological effects of GLP-1 are mediated by the binding to its specific receptor, GLP-1R [9], a G-protein coupled receptor that activates several intracellular signaling pathways [10].

Since the discovery of GLP-1, scientists have tried to translate this finding into therapies for the treatment of diabetes [11]. Unfortunately, the pharmacological effects of GLP-1 are limited by its rapid degradation. Indeed, GLP-1 is rapidly inactivated by the ubiquitous protease dipeptidyl peptidase 4 (DPP-IV). This knowledge has led to the development of several strategies, starting with the use of DPP-IV inhibitors, which may prolong GLP-1’s half-life, and moving to DPP-IV-resistant analogues, which lead to the increased activation of GLP-1R [12]. In 2005, exenatide became the first GLP-1 receptor agonist approved by the FDA to treat type 2 diabetes. It is a synthetic form of exendin-4, a homologue of mammalian GLP-1 found in the saliva of a venomous lizard (Heloderma suspectum, the Gila monster), able to bind and activate GLP-1 receptors [13]. To date, the GLP-1 receptor agonists (GLP1RA) need to be administered from once a day to once a week and have become a well-established class of glucose-lowering drugs for the treatment of type 2 diabetes [13,14].

In addition to pancreatic beta cells, GLP-1R is expressed in other tissues, including the central and peripheral nervous systems, heart, kidney, lung, gastrointestinal tract, and retinal pigment epithelium [15,16]. The wide expression of GLP-1R suggests that GLP-1 may have a broad spectrum of action, beyond the maintenance of glucose homeostasis [17]. Indeed, the clinical interest in GLP-1 analogues is also due to their effects related to a reduction in body weight. Considering the close link between overweight/obesity and type 2 diabetes, these drugs are extremely effective in the treatment of this disease. However, this action is largely independent from glucose regulation and is essentially caused by a delay in gastric emptying and reset of the hunger–satiety mechanism. However, whether there is a rise in energy expenditure is still debated.

Interestingly, the activation of GLP-1R has been found to regulate several inflammatory pathways, including oxidative stress, cytokine production, and the recruitment of immune cells in several organs [18]. Inflammation is a common element in the pathogenesis of several diseases, and its consequences are more evident in diabetic subjects, where the level of oxidative stress is increased through the high availability of glucose and its oxidation [19,20]. Contextually, hyperglycemia reduces the antioxidant defenses, further increasing the development of oxidative stress and the risk of micro- and macro-angiopathies that lead to vascular complications of diabetes [21]. In addition, oxidative stress due to hyperglycemia contributes to an increase in the release of proinflammatory cytokines, thus generating a chronic inflammatory state. The anti-inflammatory effects of GLP-1 occur through direct mechanisms, which regulate immune cells expressing GLP-1 receptors, and through indirect mechanisms, which improve glycemic control and weight loss. Although the role of overweight/obesity in inflammation is well established, it has been shown that both GLP-1RAs and DPP-IV inhibitors significantly reduce inflammation independent from weight loss or glycemic control, supporting the pleiotropic effect mediated by the binding to its specific receptor [22,23,24] and suggesting that the improvement linked to GLP-1R activation may be due to a reduction in the severity of oxidative stress. Therefore, the anti-inflammatory effects of GLP-1 may be a common mechanism through which therapies based on the use of GLP-1 analogous and GLP-1 receptor agonists, as well as DPP-IV inhibitors achieve beneficial effects, and this suggests a potential role for these classes of agents in treating other diseases. Early evidence for the anti-inflammatory proprieties of GLP-1 in humans came from the observation that treatment with the GLP-1 analogue reduced the frequency of inflammatory macrophages in individuals with type 2 diabetes mellitus [25]. Contextually, several studies have investigated the molecular mechanisms at the basis of the anti-inflammatory action of GLP-1 [18]. Considering that the activation of GLP-1R has anti-inflammatory effects in several organs, it can be hypothesized that it may also be useful in the treatment of chronic inflammatory diseases, including atherosclerosis, diabetic nephropathy, neurodegenerative disorders, nonalcoholic steatohepatitis, asthma, and psoriasis. The attention to the anti-inflammatory role of GLP-1 has recently increased, and the most recent reviews have explored the link between GLP-1, inflammation, and sepsis [26].

This Special Issue focuses on the pancreatic and extra-pancreatic anti-inflammatory effects of GLP-1, highlighting its implications not only for diabetes but also for several widespread diseases. The emerging theme of this Issue is the role of GLP-1 in neuroinflammatory disorders. This finding agrees with the increasing interest in the neuroprotective effects of GLP-1. Activation of the GLP-1 pathways also decreases the inflammatory response in several neurodegenerative disorders, including Alzheimer’s disease and Parkinson’s disease [26], which affect many people. Recently, the metabolite GLP-1 (9-36) has been found to be neuroprotective and anti-inflammatory in cellular models of neurodegeneration [27], thus showing the beneficial effects of GLP-1 in the nervous system. The tissue distribution of GLP-1 and of its receptor suggest that GLP-1 is physiologically produced near neurological targets to decrease their inflammatory state. In the brain, the primary source of endogenous GLP-1 is a population of preproglucagon neurons located in the caudal portion of the nucleus of the solitary tract near the GLP-1R expressing regions [28]. The proximity between GLP-1 production and GLP-1R expression suggests that endogenous GLP-1 may contribute to the anti-inflammatory effects in the brain. In the human retina, GLP-1 is mainly localized in the ganglion cell layer (GCL), near the inner surface [29]. In turn, the retinal ganglion cells are closely associated with astrocytes, which are responsible for the inflammation of the ganglion cell layer of the retina [30].

This evidence suggests that, in the near future, GLP-1 may be also employed in the prevention and treatment of neuroinflammatory pathologies.
